# Sweet, bloody consumption – what we eat and how it affects vascular ageing, the BBB and kidney health in CKD

**DOI:** 10.1080/19490976.2024.2341449

**Published:** 2024-04-30

**Authors:** Angelina Schwarz, Leah Hernandez, Samsul Arefin, Elisa Sartirana, Anna Witasp, Annika Wernerson, Peter Stenvinkel, Karolina Kublickiene

**Affiliations:** a Department of Clinical Science, Intervention and Technology, Division of Renal Medicine, Karolinska Institutet, Stockholm, Sweden; b Department of Translational Medicine, Nephrology and Kidney Transplantation Unit, University of Piemonte Orientale, Novara, Italy; c Department of Renal Medicine, Karolinska University Hospital, Stockholm, Sweden

**Keywords:** gut microbiome, artificial sweeteners, red meat, dysbiosis, chronic kidney disease, uremic toxins, early vascular aging, blood brain barrier, cognitive decline

## Abstract

In today’s industrialized society food consumption has changed immensely toward heightened red meat intake and use of artificial sweeteners instead of grains and vegetables or sugar, respectively. These dietary changes affect public health in general through an increased incidence of metabolic diseases like diabetes and obesity, with a further elevated risk for cardiorenal complications. Research shows that high red meat intake and artificial sweeteners ingestion can alter the microbial composition and further intestinal wall barrier permeability allowing increased transmission of uremic toxins like p-cresyl sulfate, indoxyl sulfate, trimethylamine n-oxide and phenylacetylglutamine into the blood stream causing an array of pathophysiological effects especially as a strain on the kidneys, since they are responsible for clearing out the toxins. In this review, we address how the burden of the Western diet affects the gut microbiome in altering the microbial composition and increasing the gut permeability for uremic toxins and the detrimental effects thereof on early vascular aging, the kidney per se and the blood-brain barrier, in addition to the potential implications for dietary changes/interventions to preserve the health issues related to chronic diseases in future.

In the Anthropocene, which is marked by the human impact on the environment through immense development of modern technologies, the general style and habits of people’s life are also altered accordingly.^1^ It is debatable when the influences of mankind became so big that they affected environmental factors like climate change and agricultural landscapes, however the recent development of mankind analogously resulted in societies that consume proportional high amounts of red meats, animal products and pursue low carb diets achieved through artificial sweeteners.^2^ In parallel, this is also relevant for an extensive increase and presentation of life style diseases that are developing due to complex interactions between genetic, epigenetic and functional adaptations that occur as a result of changes during the lifespan of a human and in response to environmental insults such as climate changes, food availability and potential pandemics.

Currently, it is increasingly accepted that dietary choices and practices can have a significant impact on the composition and function of the gut microbiome.^3,4^ The complex interplay between the diet and the gut microbiota influences microbial diversity, metabolic activity and overall gut health. Diets high in saturated fats have been linked to an unfavorable shift in the gut microbiome composition. This is characterized by reduced microbial diversity and an increase in potentially harmful bacteria, which can contribute to inflammation, increased gut permeability and potentially impact the development of obesity.^5^

The Western dietary pattern known for its high content of fat, salt and food additives, along with increased consumption of refined and artificial sugars and processed foods, coupled with reduced intake of whole grains and dietary fiber, not only may contribute to the development of obesity but also increase the risk of diabetes and cancer.^6^ This is also important for chronic kidney disease (CKD) patients, who are usually advised to adhere to a strict dietary plan to minimize intake of nutrients like potassium, phosphorous, sodium and proteins to help the affected kidneys that are tasked with the clearance of those from the circulation.^7^

Like many chronic diseased conditions, CKD is a multifactorial disease, influenced by many external and internal factors. The disease picture is often accompanied by uremia induced early vascular aging (EVA) phenotype and an associated increase in cardiovascular disease (CVD) risk, cognitive impairment and increased risk for depression.^8,9^ Thus, CKD patients usually must adhere to restricted diets to minimize the solute clearance burden of the functionally diminished kidneys.^7,10^ Therefore, the current health care burden requires a strong focus on dietary interventions in addition to pharmacological and renal replacement therapies, identifying and deepening the understanding how dietary aspects may influence the effects of uremic toxins on organs of vital importance.

In this review, we will concentrate on the presentation of less studied but important aspects of artificial sweeteners and red meat consumption in the gut microbiome, which may not only affect the processes of chronic disease development but also the direct effects of uremic toxins due to disturbances in microbiota composition. Further, we will elaborate on the effects of uremic toxins on kidney function and the accompanied presentation of EVA phenotype, impaired blood-brain barrier (BBB) function in CKD and how dietary interventions may improve the maintenance of a balanced microbiome and ameliorate adverse health consequences.

## Artificial sweeteners

To commence, this review addresses artificial sweeteners and red meat consumption to portray some of their impact on the gut microbiome and the consequential processes like increase in uremic toxins, before describing in more detail the associated physiological impact thereof on CKD associated EVA and their effects on the BBB.

Artificial sweeteners, widely popular as sugar substitutes, have been increasingly utilized for their apparent benefits in reducing calorie intake and managing blood sugar levels. However, emerging research has started to shine a light on the potential consequences of artificial sweeteners on the gut microbiome and kidney health. Artificial sweeteners comprising non-nutritious sweeteners (NNS) like aspartame, sucralose, saccharin or acesulfame potassium (K) and low-calorie sweeteners, which are polyols and sugar alcohols like xylitol, erythritol or sorbitol are widely used as sugar substitutes in a variety of foods, beverages and consumables. The summary of the most widely used and in the European Union authorized sweeteners is shown in Table 1.

**Table 1.** List of authorized sweeteners in the European Union according to the European Food Safety Authority, with E-numbers^11^.

E-Number	Name	E-Number	Name
**E420**	Sorbitols	**E 960a**	Steviol glycosides from Stevia
**E421**	Mannitol	**E 960c**	Enzymatically produced steviol glycosides
**E950**	Acesulfame K	**E 960d**	Glucosylated steviol glycosides
**E951**	Aspartame	**E 961**	Neotame
**E952**	Cyclamates	**E 962**	Salt of aspartame-acesulfame
**E953**	Isomalt	**E 964**	Polyglycitol syrup
**E954**	Saccharins	**E 965**	Maltitols
**E955**	Sucralose	**E 966**	Lactitols
**E957**	Thaumatin	**E 967**	Xylitol
**E959**	Neohesperidine DC	**E 968**	Erythritol

Artificial sweeteners were initially introduced as a healthy alternative due to their low or zero calorie content, which was thought to be beneficial for weight management and managing blood sugar levels. However, emerging research suggests that these artificial sweeteners cause or worsen the somatic state they were supposed to alleviate through unintended effects on, e.g. the post-ingestion pathway response and the gut microbiome.^12^ In fact, it has been shown in mice and humans that the consumption of NNS like aspartame and sucralose worsens glucose tolerance and insulin sensitivity, which are precursors to more serious conditions like metabolic syndrome and type 2 diabetes.^13^ Most artificial sweeteners are a hundred times sweeter than sucrose and used in small amounts, nonetheless they are able to activate the heterodimeric G-protein coupled sweet taste receptors taste receptor type 1 member 2 (T1R2) and taste receptor type 1 member 3 (T1R3) in the oropharynx, triggering a hormonal response, anticipatory or cephalic-phase insulin release, from the amygdala and hypothalamus in anticipation of nutrition.^14^ In the intestine, duodenal neuropod cells synapse with vagal neurons to convey sugar stimuli to the brain. Those cells are able to differentiate between sugar and sweeteners by also employing sweet taste receptors and sodium glucose transporters.^15,16^ Sugars stimulate glutamatergic neurotransmission and sweeteners stimulate purinergic neurotransmission.^15,17,18^ But artificial sweeteners are of no or little caloric value and the hormonal feedback of the post-ingestion pathway is lacking or dampened.^19^ Eventually, the lack of metabolic energy intake through the NNS dampens the hormonal response and effectiveness of satiety and energy metabolism, leading to increased sugar cravings, appetite, caloric consumption and hyperglycemia.^12,20^

Whereas low-calorie sweeteners like xylitol and erythritol, in a clinical study, have been suggested to be able to bind the taste-receptors on enteroendocrine cells since their ingestion caused glucagon-like peptide 1 (GLP-1) and incretin release, while no change in insulin secretion or gastric emptying was seen compared to the glucose control group. This implies therefore that those low-calorie sweeteners do not trigger increased appetite and are stipulated as a potential health alternative for obese and diabetic patients.^21^ Xylitol mostly passes through the small intestine and gets fermented by bacteria in the large intestine, while erythritol is mostly absorbed by the epithelial cells of the intestinal wall and then excreted by the kidneys.^22^

Several studies have suggested that artificial sweeteners can alter the composition and function of the gut microbiome.^12,23–26^ The analysis of the gut microbiome by 16sRNA sequencing of 172 NNS consuming individuals, assessed through a dietary questionnaire, showed a positive correlation between NNS consumption and taxonomic entities, including the *Enterobacteriaceae* family, the Deltaproteobacteria class and the Actinobacteria phylum.^12^ Additionally, in the same cohort, consumption of NNS was associated with increased fasting blood glucose, higher weight and waist-to-hip ratio and elevated glycosylated hemoglobin.^12^ Comparably, in male rats, a significant decrease of commensal microbes *Bifidobacterium*, *Lactobacillus* and *Bacteroides* was described after 12-week exposure to sucralose and maltodextrin.^12,27^ This indicates that repetitive consumption of artificial sweeteners can affect the microbial composition in humans and rodents.

A diverse microbial ecosystem in the gut is generally associated with good health. Some artificial sweeteners have been observed to reduce this diversity, which may contribute to conditions like obesity and diabetes. Reduced bacterial diversity can also result in decreased production of short chain fatty acids (SCFAs), which are essential for colon health.^27,28^ However, a recent clinical trial with 17 healthy patients between the ages of 18–45 suggested that daily consumption of aspartame or sucralose in doses reflective of typical high use caused no obvious differences in the microbiota community structure or in the amounts of fecal short chain fatty acids (SCFAs).^29^

In a recent study, Sihl et al. demonstrated the pathogenicity inducing effects of saccharin, sucralose and aspartame on *Escherichia coli* and *Enterococcus faecalis*, two model gut bacteria.^30^ In in vitro experiments, they showed that all three artificial sweeteners significantly increased the ability to form a biofilm for *E.coli*, whilst only aspartame had a significant effect on *E.faecalis* biofilm formation. Moreover, in co-culture with intestinal epithelial Caco-2 cells, aspartame, sucralose and saccharin, to an extent, promoted the pathogenic effects of both model bacteria. *E.coli* and *E.faecalis* showed increased adherence ability and invasion index when pre-exposed to the sweeteners, plus heightened excretion of soluble bacterial factors after sweetener exposure. Caco-2 cell viability was decreased when co-cultured with either the artificial sweeteners-exposed bacteria or addition thereof soluble bacterial factors to the culture media.^30^

In another study, Markus et al. showed that aspartame, sucralose and saccharin, commonly used sweeteners, have significant inhibitory effect on the Gram-negative bacteria N-acyl homoserine lactone-based (AHL) bacterial cell-to-cell communication system, also called quorum sensing. While not being bactericidal, the artificial sweeteners are suggested to compete with the native ligands on docking at the ligand-binding site of the AHL-receptors and thus further hinder protein folding and quorum sensing communication, affecting numerous molecular events of intestinal microbial function and subsequent the host metabolism.^31^ In another recent study, through in vitro experiments and complementary proteomic analysis, artificial sweeteners saccharin, sucralose, aspartame and acesulfame K have been shown to stimulate the transfer of antibiotic resistance genes via natural transformation in *Acinetobacter bayly*i *ADP1* and Gram-positive *Bacillus subtilis*. Additionally, artificial sweeteners could stimulate antibiotic resistance gene transfer in an in vitro model of a mouse fecal microbiome in which *B. subtilis* was added. Exposure of the bacteria to artificial sweeteners increased their cell envelop permeability, causing an upregulation of genes encoding DNA uptake and translocation machinery and prolonged plasmid resistance in transformants.^32^ This could also have an environmental impact, since artificial sweeteners to a huge extent pass through the digestive tract and urinary excretion system unaltered and the wastewater treatment plants are not equipped to retrieve artificial sweeteners properly, which causes them to end up polluting many aquatic environments.^33^ With rising antimicrobial resistance as a global public health burden, generating hundred thousands of deaths annually, a field recognized to be further investigated.^34^

Artificial sweeteners do not just affect the microbiota, they also interact with receptors of their hosts, like taste receptors, including us humans. Besides, triggering the G-protein-coupled T1R2/T1R3 heterodimer receptor in the oropharynx, it has been demonstrated that artificial sweeteners aspartame and sucralose increased intestinal epithelial barrier permeability and down-regulation of claudin 3 at the cell surface in Caco-2 cells through taste receptor T1R3. The knock-down of T1R3 in Caco-2 cells attenuated the mentioned effects of aspartame and sucralose. Additionally, claudin 3 overexpression rescued the aspartame induced reactive oxygen species (ROS) and artificial sweeteners induced barrier permeability.^35^

Artificial sweeteners appear to have the ability to affect the metabolism and the commensal gut microbiome in humans and rodents eliciting drawbacks, like increased weight, fasting blood glucose and glycosylated heamoglobin.^11,23–25 28^ Increased biofilm forming capability, enhanced antibiotic resistance gene-plasmid transfer, higher invasion index of intestinal epithelial cells and affected quorum sensing through artificial sweeteners exposure can change the balance of the gut microbiome and may cause dysbiosis, which is connected to various chronic diseases. However, it is important to note that the effects of artificial sweeteners on the gut microbiome vary depending on the type of sweetener, the amount consumed and individual differences in gut microbiome composition.^21,31,32,36^

## Artificial sweeteners and the kidneys

To date, reports on how artificial sweeteners affect the kidney are still limited and contradictive and more research is needed to investigate those effects. The consumption of artificial sweeteners may increase the workload on the kidneys as they filter out many of these compounds from the blood. Over time, this heightened workload can cause structural and functional changes in the kidneys, making them less efficient at filtering waste.^37^ Farid et al. performed an experiment with healthy BALB/c albino female and male mice, in which they were given over a period of 8 or 16 weeks for 5 h daily ad libitum access to water enriched with either sucrose, sucralose, stevia or no additive as the control group. The biochemical investigations showed that sucralose and even natural sweetener stevia significantly reduced the hemoglobin A1c level, hematocrit percentage, red and white blood cell count in male and female mice. On top of that, elevated serum levels for immunoglobulins IgG, IgE and IgA and pro-inflammatory cytokines interleukin (IL)-6 and −8 were measured in both male and female groups administered with sucralose or stevia, accompanied by a reduction in anti-inflammatory cytokine IL-10 serum level, suggesting a potential for proinflammatory effects in both sexes. Furthermore, increased urea and creatinine was measured in sucralose-exposed males and females, whereas stevia had this effect more pronounced in females. Histopathologically, no sex differences were detected in the kidney, but sucralose-exposed animals exhibited smaller sized glomeruli with enlarged Bowman’s capsules, areas of hemorrhage and loss of brush border in the proximal tubules. Stevia exposure showed a few areas of inflammation and the appearance of congested blood vessels.^38^

Interestingly, in a study by Enuwosa et al., artificial sweeteners aspartame, saccharin and sucralose are described to exhibit protective effects on the glomerular microvasculature against vascular endothelial growth factor (VEGF)-induced barrier disruption. However, their mechanistic studies using an in vitro model of primary human glomerular microvascular endothelial cells failed to identify T1R2/T1R3 activation and subsequent release of intracellular cyclic adenosine monophosphate as signaling mode of action, hypothesizing an unknown alternative signaling pathway since no sucralose was detected within the cytosol of glomerular endothelial cells.^39^ Further, no major effects of saccharin on the glomerular filtration rate (GFR) or urine flow in the kidney were seen in an in vivo experiment on 30 male Wister rats, which have been administrated with an acute saccharin infusion, suggesting that any reported change in renal function with artificial sweeteners must depend on chronic consumption.^40^

Aspartame has been described as a nephrotoxin, its metabolites phenylalanine, aspartic acid and methanol, which gets metabolized to formaldehyde, have detrimental effect on the kidney and cause oxidative stress.^41–43^ Daily oral administration of aspartame in male Wistar rats over a period of 42 days resulted in increased urea, creatinine and potassium in serum, while blood hemoglobin, sodium and calcium levels were significantly decreased. Additionally, aspartame treatment decreased glutathione and the activities of glutathione peroxidase and catalase in kidney tissue with a simultaneous increase of thiobarbituric acids reactive substances, known as a byproduct of lipid peroxidation and increased oxidative stress. Co-treatment with folic acid and N-acetyl cysteine alleviated the observed effects of aspartame in the Wistar rats.^43^ In a different study, female albino Wistar rats were through orogastric administration exposed on the 9th, 10th and 11th day of pregnancy to either at room temperature or heated to 40°C dissolved aspartame. Heating causes aspartame to form free phenylalanine and diketopiperazine, seemingly a carcinogen. The experiment showed that the administration of aspartame during pregnancy led to alterations in the fetal renal structure, stereological parameters showed significantly increased cell volume and decreased numerical cell density in the tubules and glomeruli of the aspartame exposed fetal kidneys for both temperature conditions.^44,45^

In summary, increasing evidence suggest that, at least based on animal experiments, the usage of artificial sweeteners has an effect on the kidney through structural changes in the glomeruli and congested blood vessels, on inflammation, blood hemoglobin, sodium and calcium levels.

**Figure 1.**
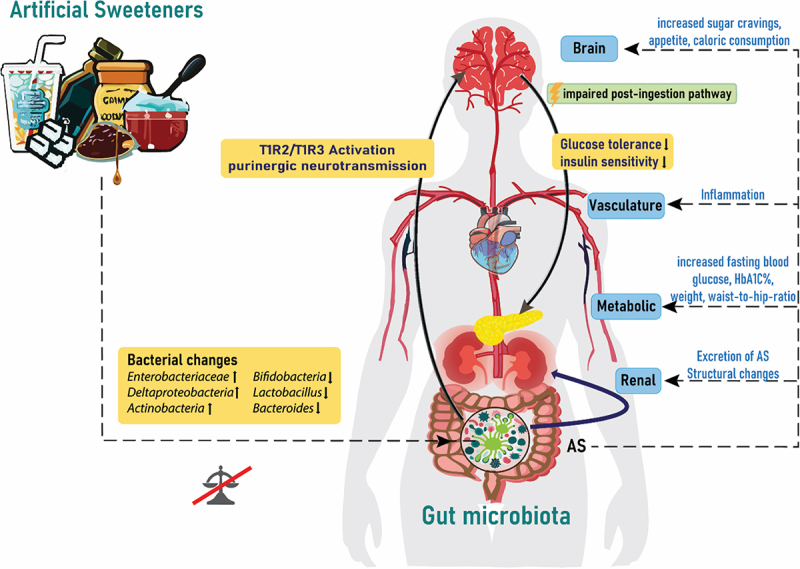
Artificial sweeteners affect the gut microbiota and endocrine response. Artificial sweeteners from different dietary products can affect the microbial gut composition via activation of taste receptors linked to the modulation of, eating behavior, glucose tolerance and handling insulin sensitivity and inflammation. AS: artificial sweeteners; T1R2/T1R3: taste receptor type 1 member 2 and 3; HbA1C%: glycosylated hemoglobin.

However, the evidence of how this affects sugar control is contradictory based on levels of glycated hemoglobin for some of the artificial sweeteners. Figure 1 illustrates the interplay of artificial sweeteners with the gut microbiome and the following endocrine responses.

## Red meat and uremic toxins

The planet’s consumption of red meat has doubled since the 1960s and above all, it is in China that the consumption of red meat is increasing.^46^ The dramatic increase in red meat consumption not only increases greenhouse gas emission but will also impact our health.^47,48^ Robust scientific evidence links increased consumption of red meat intake with increased risk for CVD as well as metabolic syndrome, type 2 diabetes, diverticulitis and cancer, leading to a negative perception of the role of high intake of red meat for health.^49–51^ High consumption of red meat results in an increased intake of saturated fat, cholesterol, iron and salt, as well as an excessive acid load. Although red meat is the best source of essential amino acids that are more nutritionally efficient than are those provided by vegetables, recent data suggest that red meats have multiple negative effects on our health. Red meat is rich in sodium and phosphate and can promote acidosis.^49^ Consumption of red meat in humans is also associated with an elevation in inflammatory biomarkers and the promotion of oxidative stress.^52^

The influence of diet on human health is mediated at least in part by the gut microbiota. Gut dysbiosis is understood as the altered composition and function of the gut microbiome, which refers to the community of microorganisms, including bacteria, viruses and fungi, which live in our digestive tract. These commensal microbes play a crucial role in our health, aiding in digestion, immune function and even mental health.^23^ The entrance of bacteria into the blood circulation can be seen as an intrinsic factor of gut leakiness and may reflect the gut health.^53^ Increasing evidence suggests that different socioeconomical positions may play an important role in gut microbial composition. For example, when analyzing the peripheral venous blood using 16SRNA sequencing, differences in the circulating microbiota were seen in the Glasgow community groups depending on their socioeconomical status linked to poor nutrition and accelerated biological aging.^54^ Indeed, those that were characterized as most biologically aged exhibit a significantly higher abundance of circulatory pathogenic bacteria, including *Neisseria* and *Porphyromonas*, while those less biologically aged possess more circulatory salutogenic or commensal bacteria, including *Lactobacillus*.^54^

A recent review of 85 eligible articles concludes that there is a paucity of research in this area and that the directionality and magnitude of changes in the gut microbiome varied with inconsistent pattern.^50^ Thus, although red meat intake tended to increase the population of some bacterial species and decrease population sizes of other genera, however robust data are lacking. Part of the inconsistencies in the literature could be linked to the fact that “meat” is not a single food but includes a wide variety of muscle food from mammalian, aquatic, avian and other sources of flesh and other tissue food. Even though there are varying reports about bacterial changes, the food source affects the production of bacterial metabolites like uremic toxins. It was recently reported that red meat shifts the gut microbiome to produce more trimethylamine (TMA), the precursor of trimethylamine-N-oxide (TMAO) and a uremic toxin, via a microbial gene cluster.^49,55^ Dysbiosis in turn compromises intestinal barrier function, making it more permeable to an influx of bacterial fragments from gut to blood.^56^ By analyzing microbial DNA signatures within the blood circulation, we recently confirmed that TMAO along with one carbon metabolism had significant impact upon both inflammatory burden and the composition of the microbiome in CKD.^57^ Our findings demonstrate that TMAO acts as the key toxin shaping the uremic microbiome and therefore this polyamine might be exploited to enable dietary intervention strategies that restore the microbiome in CKD.

High consumption of animal protein sources, especially red meat, increases the production of several uremic toxins, including p-cresyl sulfate (PCS), indoxyl sulfate (IS) and TMAO.^49,57^ Although the physiological impact of this remains to be fully elucidated, a lot of interest has been focused on TMAO, which appears to be an underlying feature of inflammatory diseases associated with aging.^58^ Red meat, compared to other protein sources, is specifically high in substrates such as carnitine that promote TMAO production. Emerging evidence suggests that frequent red meat consumption may also speed up the aging process.^59^ In CKD, impaired solute clearance causes their accumulation in the body, resulting in a condition called “uremia”. Uremia occurs when the kidneys are unable to eliminate metabolic waste products known as uremic toxins, leading to retention of substances that are normally excreted in urine.^60^ Uremic toxins have been implicated in the disruption of the intestinal wall barrier, leading to increased permeability. Individuals with renal dysfunction often end up in a state of dysbiosis, where the altered composition of gut microbiota produces an abundance of uremic toxins that accumulate in the bloodstream and further harm the vasculature and the kidneys.^36,61^ Gut derived uremic toxins were shown to negatively impact vascular health by inducing endothelium and smooth muscle cell dysfunctions through the modulation of oxidative stress, inflammatory responses and cytokine activity.^62,63^

Even though numerous uremic toxins have been identified and many are believed to contribute to the progression of CKD and cardiovascular disease, only a few have been thoroughly investigated. Most studies fail to consider the potential synergistic effects of multiple toxins on organ damage, despite it being likely that a combination of various toxins contribute to the development of complications in CKD patients. While much research has focused on the systemic effects of uremic toxins, the following review’s chapters will concentrate on their detrimental effects on the vasculature and BBB.^64–66^

## Uremic milieu and EVA phenotype

The uremic milieu that accompanies CKD has been linked to a clinical model of premature aging, which is characterized by persistent low-level inflammation, muscle loss, osteoporosis, frailty and a significantly higher risk of cardiovascular mortality.^67,68^ Individuals with end-stage kidney disease (ESKD) have a cardiovascular mortality rate above ten times higher than that of the general population.^69^ Although the precise pathological mechanisms leading to the heightened cardiovascular mortality are not fully understood, the presence of EVA appears to be one of the primary factors.^8^ The dissociation between chronological and biological age in individuals with uremia is a typical feature of EVA. The buildup of uremic toxins contributes to an overall increase in allostatic load, the cumulative wear and tear on the body’s physiological systems due to chronic stress, which can accelerate the aging process and contribute to the development of EVA.^70^

Gut-derived uremic toxins like IS and PCS can promote inflammation, oxidative stress and endothelial dysfunction, which are key contributors to EVA.^71^ The toxins activate inflammatory pathways and increased production of ROS, which leads to damage of the endothelial cells lining the blood vessels and impairment of the normal regulation of vascular tone, promoting the formation of atherosclerotic plaques and increased cardiovascular risk. Uremic toxins can directly affect the structure and composition of the arterial wall, promoting collagen deposition and calcification, which contribute to the development of arterial calcification and stiffness, a hallmark of EVA.^72^

Abnormalities in the regulation of calcium and phosphate play a crucial role in the maintenance and development of vascular calcification.^73^ At a cellular level, advanced glycation end-products, PCS, IS, TMAO, phosphatidylinositol (Pi) and phosphaturic hormones facilitate endothelial dysfunction and phenotypic changes in vascular smooth muscle cells (VSMCs).^74,75^ Notably, dysbiosis coherent with increased uremic toxin production can contribute to vascular calcification as gut microbiota are the most significant source of PCS, IS and TMAO.^76^

## Trimethylamine-N-oxide (TMAO)

So far, the precise role of TMAO in the development of calcification and its mechanisms contributing to vascular calcification are not fully understood. Zhang et al. conducted a study using a rat model, confirming that TMAO induces vascular calcification by causing a dose-dependent increase of calcium in VSMCs cultured in a calcifying environment. TMAO additionally stimulated the expression of runt-related transcription factor 2 (Runx2) and bone morphogenetic protein 2 (BMP2), genes involved in the osteoblastic differentiation of VSMCs. Furthermore, in both in vivo and ex vivo experiments, TMAO led to an accumulation of mineral content and upregulated genes responsible for the transdifferentiation of VSMCs into osteoblast-like cells. In the same study, TMAO increased the serum levels of IL-1β of CKD rats and caused activation of the NLR family pyrin domain containing 3 (NLRP3) inflammasome and upregulation of nuclear factor kappa B (NF-κB), both factors involved in the transcription of IL-1β.^77^ Another study by Yazdekhasti et al. in mice lacking apolipoprotein E (apo-E) suggested a possible role of TMAO in atherogenesis. Interestingly, a diet based on fish-derived protein resulted in more advanced atherosclerosis in the aorta with increased calcification of atherosclerotic lesions. The concentration of TMAO was approximately six times higher in mice receiving the fish-based protein diet, suggesting that TMAO originating from fish protein metabolism may be the key factor responsible for these observed differences.^78^ Whereas human studies have produced conflicting results. One observational study involving 4,007 patients undergoing elective coronary angiography found a relationship between fasting plasma levels of TMAO and the occurrence of major adverse cardiovascular events (death, myocardial infarction or stroke) over a three-year follow-up period.^79^ This study, however, did not investigate the prevalence and progression of vascular calcification. Further, the Coronary Artery Risk Development in Young Adults (CARDIA) study did not find any influence of TMAO on the onset or progression of coronary artery calcification score or common carotid artery intima-media thickness during a ten-year follow-up period.^80^ However, additional reports revealed detrimental effects related to high serum TMAO levels, specifically in white patients on dialysis, without the same effect being observed in black patients.^81–83^

Studies have indicated that elevated levels of TMAO consequently accelerate the progression of kidney dysfunction by influencing the development of tubular-interstitial fibrosis and deposition of collagen, likely through increased activation of Smad3 and downstream target genes.^84,85^ Smad3 is known as a critical mediator of transforming growth factor-β1 (TGFβ1) signaling and plays a driving role in both renal inflammation and fibrosis.^86,87^ Findings from meta-analyses and systematic reviews have linked TMAO to an increased risk of hypertension, as well as adverse cardiovascular events and all-cause mortality across the general population, elderly individuals and patients with CKD.^88–91^ Recently, Bang-Gee H et al. reported that TMAO may serve as a significant upstream regulator in the development of peripheral arterial stiffness among individuals in advanced CKD stages 3–5.^92^ Inhibition of TMAO using choline TMA-lyase mechanism-based inhibitor, iodomethylcholine, in a murine model of CKD has been found to reduce serum cystatin C levels, as well as alleviate the severity of renal tubular-interstitial fibrosis and collagen deposition.^93^ In rats with 5/6 nephrectomy, TMAO along with superoxide and proinflammatory cytokines was significantly elevated, while endothelial nitric oxide production was reduced, collectively contributing to endothelial dysfunction.^64^ A study conducted by Brunt et al. revealed a positive correlation between plasma TMAO levels, aortic pulse wave velocity and systolic blood pressure in aging humans and mice.^94^ Furthermore, they indicated that TMAO has detrimental effects on intrinsic mechanical stiffness by influencing the formation of advanced glycation end-products and ROS. This study yielded findings showing that CKD patients without diabetes mellitus, regardless of hypertension or hyperlipidemia, had a significantly higher risk of peripheral arterial stiffness in relation to TMAO levels. However, in diabetic CKD patients, TMAO did not correlate with arterial stiffness, suggesting a potential influence of diabetes mellitus in modulating the role of TMAO in peripheral arterial stiffness.^94^

## Indoxyl phosphate (IS)

IS, a potent uremic toxin, has been documented for its negative impact on kidneys and the vascular system playing a role in endothelial dysfunction and facilitating inflammation. IS accumulates in the circulation as a byproduct of dietary tryptophan metabolism by gut bacterial tryptophanases and the indole pathway.^60,95^ After being produced by intestinal bacteria like *E.coli*, *lactobacilli* or *Bacteroides fragilis*, indole is absorbed into the portal circulation and transported to the liver where indole undergoes hydroxylation mediated by cytochrome P450 2E1 (CYP2E1) resulting in the formation of 3-hydroxy indole.^96,97^ Following hydroxylation, indole is further metabolized by sulfotransferase 1A1 (SULT1A1), which catalyzes the sulfation of indole to produce IS. IS is involved in the pathophysiology of cardiovascular complications and has not only been linked to CKD fibrosis progression but also to changes in bone-mineral metabolism, insulin resistance and development of anemia. Several in vivo and in vitro experiments have described the induction of vascular calcification caused by IS toxicity.^98–100^ Among its various mechanisms to trigger vascular calcification, IS enhances oxidative stress, upregulates the expression of transcription factor NF-κB, involved in inflammation and downregulates transcription factor nuclear erythroid 2-related factor 2 (Nrf2), involved in anti-oxidative defense.^101^ Epigenetically, IS has been suggested to influence microRNAs that regulate the transdifferentiation of osteoblastic VSMCs.^102^ Zhang and colleagues demonstrated that IS downregulates miR-29b, a suppressor of vascular calcification, in radial arteries from ESKD patients and human aortic VSMCs.^77^ In a CKD mouse model, Nakano et al. found that IS can accelerate atherogenesis and vascular calcification by stimulating proinflammatory macrophages.^98^ Opdebeeck et al. showed that both IS and PCS induce vascular calcification in rats with adenosine-induced kidney injury when reaching serum concentrations comparable to those observed in CKD patients.^99^ Experimental models of chronic kidney injury have demonstrated that IS and PCS can activate the renin-angiotensin-aldosterone axis, upregulate angiotensin II type 1 (AT1) receptors and downregulate angiotensin II type 2 (AT2) receptors, which are stipulated to have similar effects on vascular damage, remodeling and potentially vascular calcification.^63^ Shimizu et al. discovered that IS, at concentrations similar to those found in CKD patients, potentiates the detrimental effect of angiotensin II (AngII) on VSMCs in CKD rats, primarily through IS-induced oxidative stress.^103^ CKD patients with higher serum IS levels are prone to aortic calcification and high mortality, suggesting that IS acts as a procalcifying toxin.^104^

Furthermore, in vitro and in vivo studies demonstrated that IS-treated human aortic smooth muscle cells could promote aortic calcification and aortic wall thickening and enhance the expression of osteoblast specific proteins, senescence and calcification due to oxidative stress resulting from nicotinamide adenine dinucleotide phosphate (NADPH) oxidase upregulation.^105,106^ AST-120 is an orally administered drug and works as an intestinal absorber of IS, reducing the level of IS in blood and urine, improving uremic symptoms.^107^

In vitro studies have revealed that endothelial cells incubated with IS exhibited impaired proliferation, delayed wound repair, reduced nitric oxide production, increased cell senescence and heightened oxidative stress, all of which indicate the adverse effects of IS on endothelial function.^108^ Studies on CKD patients have demonstrated negative correlations between baseline IS levels and vascular reactivity index, as well as a positive association between IS levels, carotid-femoral pulse wave velocity and aortic calcification.^104,109^ Earlier research has demonstrated a significant positive correlation between serum IS and aortic calcification in CKD patients.^110^ This phenomenon has also been observed in hypertension rat models, where it was further demonstrated that the aortic wall thickened and showed an increased expression of osteoblast-specific proteins.^111^

## p-cresyl sulfate (PCS)

PCS, another uremic toxin, that is produced by gut microbiota like *Bacteroides fragilis* and *Bacteroides caccae*, which increases in serum levels as GFR decreases, has been associated with cardiovascular damage in CKD.^96^ In a CKD rat model, Opedebeek et al. showed that sustained exposure to PCS or IS leads to significant calcification in the aorta and peripheral vessels, ranging from moderate to severe levels. Either PCS or IS was administered through the drinking water with a concentration set to achieve a daily intake of 150 mg/kg starting from week 3 until euthanasia at week 7. The excess calcification primarily accumulated in the media layer, when observed in cross-sections of calcified vessels. Both IS and PCS were found to promote the migration and proliferation of VSMCs, which are crucial cellular events in the development of vascular calcification.^99^ Exposure to PCS of atherosclerosis-prone mice lacking ApoE on a high-fat diet resulted in heightened formation of atherosclerotic plaques compared to control animals. Furthermore, PCS disrupted the balance between matrix metalloproteinases and tissue metalloproteinase inhibitors, contributing to plaque instability. Gross et al. conducted an animal study in mouse thoracic aorta, revealing that PCS triggers oxidative stress in endothelial cells and VSMCs and it induces the contraction of smooth muscles in the aortic wall when exposed to phenylephrine. This process leads to inward eutrophic remodeling of the aortic wall, a sign of uremic vasculopathy, which is characterized by the reduction of the area of both lumen and media.^112^

Serum PCS has been identified as a predictor of arterial stiffness in patients undergoing hemodialysis.^113^ As renal function declines, PCS progressively accumulates and is considered a detrimental factor in renal fibrosis due to its ability to increase the production of ROS, activate TGFβ, stimulate the renal-angiotensin-aldosterone system and induce renal tubular damage.^63,114,115^ PCS has also been associated with image-proven vascular calcification and carotid-femoral pulse wave velocity, while showing an inverse relationship with estimated (e)GFR in CKD patients.^116^ Recently, Opdebeeck et al. demonstrated that short- and long-term exposure to PCS promotes aortic inflammation and calcification, respectively, through the acute-phase response and coagulation signaling pathway.^99^ In a cross-sectional study, Rossi et al. reported that serum PCS was independently associated with IL-6 and pulse wave velocity, highlighting its role in inflammation and its contribution to cardiovascular damage in CKD stages 3–4.^117^ PCS has been linked to endothelial dysfunction, arterial stiffness, vascular calcification, cardiovascular events and all-cause mortality in patients with CKD and on hemodialysis.^108,116,118^

## Phenylacetylglutamine (PAG)

**Figure 2.**
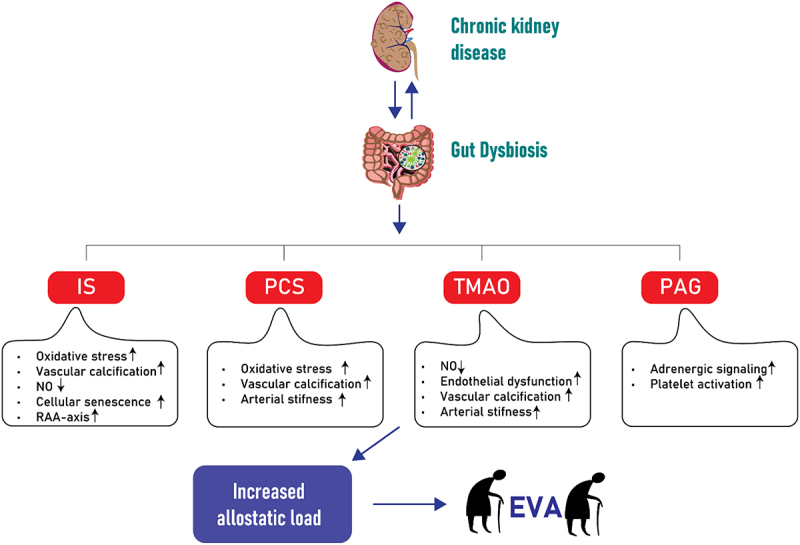
Simplified illustration of uremic toxins, specifically IS, PCS, TMAO and PAG and their association with increased allostatic load and early vascular aging (EVA) in the context of CKD. The diagram outlines the pathways through which these toxins contribute to the increased EVA with a focus on major impact related to vascular function and structure, such as endothelial dysfunction, calcification, cellular senescence and arterial stiffness among others. IS: Indoxyl sulfate; PCS: p-cresyl sulfate; TMAO: Trimethylamine N-oxide; PAG: Phenylacetylglutamine; CKD: chronic kidney disease; EVA: early vascular aging; RAA: renin-angiotensin-aldosterone; NO: Nitric oxide.

Another microbial toxin that has gained attention is PAG, a colonic microbial product resulting from the metabolism of dietary phenylalanine. In PAG synthesis gut bacteria transform dietary protein-derived phenylalanine into phenylpyruvic acid through widespread deamination, facilitated by microbial enzymes like phenylalanine dehydrogenase and aromatic amino acid aminotransferase.^119,120^ Gut microbes then convert phenylpyruvic acid into phenylacetic acid.^66^ After being absorbed into the portal circulation, hepatic and renal enzymes of the host catalyze the conjugation of phenylacetic acid with either glutamine, resulting in the formation of phenylacetylglutamine (PAGln), or glycine leading to the formation of phenylacetylglycine (PAGly).^66^ Symbiotic gut microbes in humans and vertebrates, capable of producing phenylacetic acid, have been identified in bacteria isolates as *Bacteroidetes*, *Firmicutes* and *Proteobacteria*.^66,121–124^ Notably, Poesen et al. reported that PAG is linked to overall mortality and CVD in patients with CKD.^125^ With systematic series of genetic loss-of-function studies and gain-of-function studies, as well as multiple corroborative pharmacological inhibitor and agonist studies, using human embryonic kidney cells and platelets, Nemet et al. demonstrated that PAGln signals through α2A, α2B and β2 adrenergic receptors that are expressed on platelets and linked to platelet activation toward thrombosis, ultimately suggesting their potential involvement in CVD. Their studies indicated a saturable and specific binding of PAGln to cells, indicative of a cell receptor – ligand interaction.^66^ In another study, Liu Y et al. found an independent association between plasma PAG levels and coronary atherosclerotic burden in patients with suspected coronary artery disease.^126^ Additionally, plasma PAG levels were shown to be associated with an increased risk of incident coronary artery disease and peripheral artery disease.^127^ Not confined to CVD patients, clinical studies have established independent associations between PAG and CVD in the general population, as well as a link between PAG, CVD and mortality in patients with CKD.^125,127^ Understanding the association between gut-derived uremic toxins and EVA is of great importance, as it highlights the potential role of the gut-kidney axis in cardiovascular health. Targeting the gut microbiota and reducing the production and absorption of these toxins may offer therapeutic opportunities to slow down or prevent EVA and its associated cardiovascular complications in individuals with kidney dysfunction. In figure 2 a schematic overview illustrates the above discussed uremic toxins and their association with EVA in the context of CKD.

It is well recognized that the gut microbiome constitutes a mechanistic link between CKD and uremic inflammation.^128^ Gut-derived uremic toxins, including IS, PCS, TMAO and PAG accumulate when kidney function declines, activating pro-inflammatory and pro-fibrotic pathways.^60^ On top of this, functional and metabolic changes of the gut microbiota contribute to the age-associated chronic, low-grade inflammation termed inflammaging, which is implicated in the pathogenesis of age-related diseases.^129^ Inflammaging, along with oxidative stress, genomic damage, cellular senescence and imbalanced pro-aging and anti-aging systems are suggested drivers of the premature aging phenotype, which is typical of CKD patients and associated with high risk of cardiovascular complications, muscle wasting, osteoporosis and frailty.^130–132^ Thus, the microbiome is potentially involved both in the progression of CKD as well as CVD, as inflammatory markers that are shown to predict CKD progression are to a large extent also associated with CVD risk.^60, 133–137^

## Uremia, gut microbiota and CNS implications

**Figure 3.**
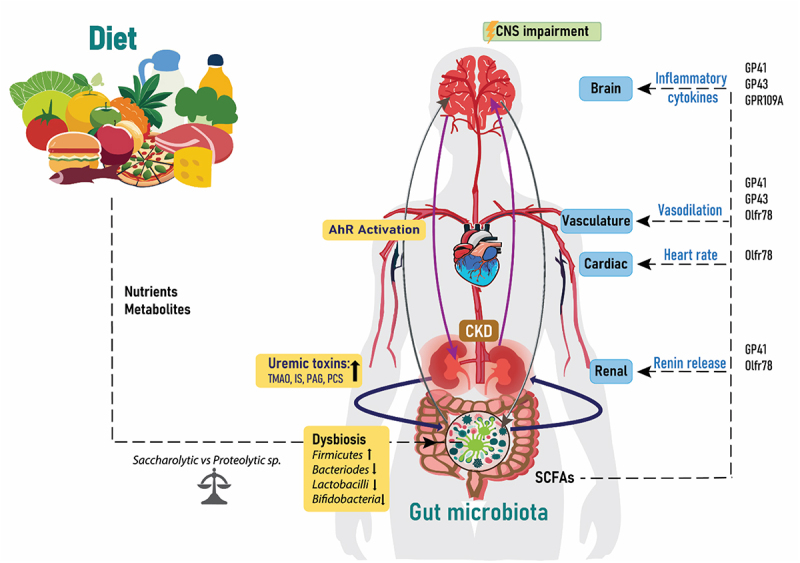
Diet, gut, brain and CKD. The relationship between the gut microbiota, kidney and brain is a circulatory process, which is influenced by the nutrients and metabolites from dietary choices. Increased bacterial changes lead to dysbiosis, which increases the release of uremic toxins into the circulation and activation of the AhR receptors and thereafter effects on the CNS. The amount of SCFAs from the gut microbiota influence renal renin release, heart rate and vasodilation and inflammatory cytokines through acting on the listed proteins. IS: Indoxyl sulfate; PCS: p-cresyl sulfate; TMAO: Trimethylamine N-oxide; PAG: Phenylacetylglutamine; CKD: chronic kidney disease; SCFAs: short chain fatty acids; CNS: central nervous system and AhR: aryl hydrocarbon receptor.

In recent years, the scientific community has acknowledged the concept of the connection between the uremic environment, gut microbiota and the brain, also termed the “microbiota-gut-brain-axis”.^138,139^ As the understanding of the complex relationship between microbiome, gut, central nervous system (CNS) and CKD has expanded, it could be fitting to conceptualize this as the “microbiota-gut-kidney-brain axis” (Figure 3).^138,140^ In this axis, gut dysbiosis results in lower levels of saccharolytic microbes and increase in proteolytic microbes, producing metabolites and toxic substances associated with the uremic environment.^141^ As described above, gut microbiota are involved in the metabolism of dietary tryptophan into indole derivatives, like uremic toxin IS, which can activate aryl hydrocarbon receptor (AhR), a receptor for multiple physiological ligands.^142^ The damaging effect of uremic toxins on the vasculature and the impaired renal clearance in CKD patients leads to further accumulation of these toxins in the circulation, which can impair the BBB integrity.^96, 143–145^

The gut microbiome and the CNS establish communication via immune, endocrine and neural pathways.^146^ The two-way communication between the gut and CNS is tightly regulated at important interfaces such as the intestinal epithelium and the BBB.^147^ Enteroendocrine cells, enterochromaffine cells and gut associated immune cells facilitate transmission of signals from nutrients, microbial metabolites, toxins and irritants from the gut to the CNS.^147^ This bidirectional communication extends even to the prenatal period, where the maternal gut microbiome exerts influence on fetal neurodevelopment through the bacteria *Clostridiales*, which is able to produce SCFAs that can enter the BBB.^148^ Additionally, *Escherichi*a and *Shigella*, members of the proteobacteria phylum, which dominate the neonatal gut, influence neurodevelopment through various mechanisms including direct communication via vagal nerves, production of microbial metabolites, cytokine production and BBB interaction via their lipopolysaccharides eliciting immune response.^148^ This highlights the far-reaching impact of the gut microbiota not only in adult life but also during crucial developmental stages early in life.

As mentioned in earlier sections of this review, the gut microbiota composition is significantly influenced by dietary factors, with artificial sweeteners emerging as potential contributors to alterations in microbial abundance and diversity. This influence potentially disrupts the balance of the microbiota-gut-brain axis, which then contributes to changes in metabolite production and toxin release, affecting the overall homeostasis of the microbiome and consequently the CNS. A rodent animal study has shown that bacterial metabolites like SCFAs possess the capacity to regulate the maturation and function of microglia, hence affecting brain health.^149^ This interconnection, illustrated in Figure 3, is influenced by dietary choices leading to dysbiosis, increased release of uremic toxins, activation of AhR receptors and subsequently repercussions for the CNS.

In the intestine, sensory neurons form connections with motor neurons, influencing the regulation of intestinal motility and secretion of gut hormones.^150^ Gut dysbiosis has been associated with a range of diseases and disturbances in physiological functioning.^151^ For example, alterations in the gut microbiota have been associated with neurological disorders such as autism spectrum disorders, depression, anxiety and neurodegenerative diseases.^146,152,153^ Notably, male and female patients with major depressive disorders have been observed to have higher *Actinobacteria* and lower *Bacteroidetes* levels.^154^ CKD patients have a greater burden and risk of experiencing depressive disorders and cognitive impairment, which is associated with adverse outcomes.^155–158^ The increased levels of pro-inflammatory cytokines and altered immune response in CKD, coupled with gut dysbiosis, may further contribute to neuroinflammation and neurodegenerative processes.^159,160^

In addition, gut microbes play a crucial role beyond intestinal functions. They not only influence the BBB permeability but also affect the hypothalamic-pituitary axis activity and vagus nerve stimulation.^161–164^ This results from the production of both excitatory and inhibitory neurotransmitters and chemical signals. Certain gut bacteria have the capacity to produce and metabolize neurotransmitters such as serotonin, dopamine, y-aminobutyric acid (GABA), histamine and acetylcholine.^165,166^

Serotonin, implicated in emotional distress and irritable bowel syndrome can be produced by bacterial strains like *Streptococcus spp., Enterococcus spp., Escherichia spp., Lactobacillus spp, Lactococcus spp, Klebsiella pneumonia* and *Morganella morganii*. Similarly, dopamine production has been attributed to *Escherichia, Bacillus, Lactococcus, Lactobacillus* and *Streptococcus*.^167–169^ GABA, can be efficiently produced from strains of *Lactobacillus* and *Bifidobacterium*, highlighting the probiotic potential of the gut bacterial strains *Lactobacillus rhamnosus* and *Bifidobacterium longum* in alleviating stress, anxiety, depression and withdrawal symptoms by aiding restoration of GABAergic activity in the brain.^163,170–172^ These neurotransmitters, known regulators of mood, behavior and cognition underscore the gut microbiota’s significance in influencing the CNS.^165,170,173^

The impact of the bioactive molecules, including neurotransmitters, SCFAs and metabolites, produced by gut microbes is not limited to peripheral effects but can directly or indirectly impact the CNS by crossing the BBB, modulating neural activity and neuroinflammation.^174^ Germ-free mice studies established the regulatory role of gut microbiota in BBB permeability, when upon re-introduction of gut microbiota BBB integrity improved and an up-regulation of tight junction protein expression was seen.^175^ AhR agonists such as IS, have the ability to cross the BBB indicating their important role in influencing and regulating the CNS function. ^136,176^ In CKD, where uremic toxins such as IS contribute to inflammation, metabolic dysregulation and microvascular dysfunction, understanding the sex-specific differences in AhR expression and their relationship to BBB permeability is increasingly becoming relevant.^177–179^ Moon and colleagues have reported disparities in BBB integrity between males and females.^180^ An animal study showed 150% higher AhR mRNA expression in female rats compared to males.^181^ Whilst Navar et al. reported a downregulation of AhR expression in aged female macrophages, coupled with an increase in phagocytic activity in estrogen treated cells, suggesting sex-differences in immune responses and inflammation.^182^ Thus, in the context of CKD, where inflammation plays a crucial role in disease progression, such findings may explain one of the factors influencing sex differences observed in CKD.

Traditional risk factors in CKD such as hypertension, diabetes, age, smoking, sex and family history intertwine with non-traditional risk factors including gut dysbiosis, uremic toxins, environmental stress, malnutrition, persistent inflammation, physical inactivity and somatic stem cell mutation, which all contribute to the multifactorial nature of CKD complications.^130^ Metabolites like SCFAs produced by the gut microbiota, play a regulatory role in blood pressure through interactions with host G-protein-coupled receptors including GP41, GP43, GPR109A and olfactory receptor (Olfr) 78 in mice and OR51E2 in humans.^183,184^ SCFAs traverse the circulation, stimulating receptors in the kidney to boost release of renin, activating inflammatory cytokines and pressure response in the brain, influencing vasodilatation and heart rate.^185^

The link between CKD and gut dysbiosis is considered a double-edged sword, the progression of CKD is influenced by gut-derived metabolites and toxins, simultaneously, the gut microbiota composition is affected by the uremic environment.^186^ One study investigated the gut microbial composition across various CKD stages and observed variations in bacterial abundance from CKD 1 to CKD 5.^187^ The abundance *of Butyricicoccus spp, Clostridium difficule, Enterobacteriaceae, Escherichia coli, Lactobacillus spp, Roseburia spp* and *Streptococcus spp* differed across different CKD stages and control group.^187^ Additionally, the researchers identified gut bacteria capable of producing uremic toxins. The study noted several bacterial species from diverse genera including *Bacteroidaceae, Clostridiaceae, Lachnospiraceae, Staphylococceae, Actinomycteae, Tannerellaceae, Enterococceae, Bacillaceae, Bifidobacteriaceae, Brevibacteriaceae, Coriobacteriacee, Corynebacteriaceae, Eggertheraceae, Microbacteriaceae* and *Micrococceae* capable of generating uremic compounds. Further, protein-bound uremic toxins in CKD patients were primarily produced by anaerobic bacteria, while both anaerobic and aerobic bacteria contributed to the production of indolic compounds.^187^ The EQUAL study identified specific uremic toxins linked to symptoms such as fatigue, constipation and shortness of breath, revealing sex-based variations.^188^ TMAO was associated with fatigue and PCS and PAG were linked to constipation with notable differences between males and females. Specifically, higher levels of TMAO, PCS and PAG were associated with symptoms in males compared to females.^188^

The relationship between CKD and gut microbiota may be influenced by factors such as nutritional elements, medications and treatment strategies. CKD patients that are undergoing renal transplantation also have a substantial immunosuppressive therapy and antibiotic treatment to prevent infection, thus potentially triggering significant changes to their gut microbiota. While the topic on renal transplantation is indeed expansive, understanding the changes in microbiota diversity in renal transplant patients may offer insight into its broader implications in patients’ overall health.^189^ The gut microbiome composition in kidney transplant recipients notably differs from healthy individuals with pretransplant patients showing abundant *Firmicutes*, while post-transplant patients exhibit higher levels of Proteobacteria (*Escherichia coli*) and lower Actinobacteria (*Bifidobacterium* species).^190,191^ Notably, patients experiencing diarrhea post-kidney transplantation have been observed to lack certain beneficial bacterial species such as *Bacteroides, Ruminococcus, Coproccus* and *Dorea* species.^190^ While the direct link between microbiota changes and uremic toxins remains to be fully understood, recognizing that there is an interplay between the two, highlights the complex nature of CKD and its impact on gut health.

CKD patients are prone to various complications like bone diseases, insulin resistance, renal fibrosis, CVD and brain-related conditions. Focusing on the latter, CKD-related CNS disorders may manifest as altered mental status based on two proposed hypotheses: the vascular hypothesis and the neurodegenerative hypothesis. The vascular hypothesis attributes CNS disorders to endothelial dysfunction influenced by both traditional and non-traditional risk factors. In contrast, the neurodegenerative hypothesis advocates the direct or indirect contribution of uremic toxins to CNS injury during CKD development, involving circulating toxins that pass and impair the BBB, affecting the brain.^192^

In relation to the neurodegenerative theory, toxins can alter the secretion of neurotrophins, including brain-derived neurotrophic factor (BDNF).^193^ BDNF is produced by different cell types in the CNS, such as neurons, glial cells and microglia and it is vital to the processes of neurogenesis and differentiation, as well as neuronal plasticity essential for long-term memory.^194^ In a study involving germ-free mice, diminished BDNF levels were observed in the hippocampus and cortex brain areas compared to controls, which suggests a modulating involvement of the gut microbiome.^195^ Lower BDNF levels are also noted in CKD patients and individuals, especially females with major depressive disorder, compared to healthy controls.^196,197^ Furthermore, BDNF levels increase after kidney transplantation.^198^

It is worth noting that in patients with renal impairment, sleep quality can affect their physical and mental well-being and ultimately their overall quality of life. ESKD or CKD-5 patients have higher prevalence of poor sleep quality compared to those with CKD stages 1–4.^199,200^ Additionally, females and those undergoing dialysis also tend to have lower sleep quality.^199,200^ Insufficient sleep has been linked to metabolic disorders like obesity, diabetes, CVD and neurological and cognitive impairments and even cancer.^201,202^ Meanwhile, alterations in gut microbiome composition have been observed in individuals with these pathologies.^203,204^ Sleep quality and dietary practices can have a significant impact on the composition and function of the gut microbiome.^3,4^ The Mediterranean diet, typically rich in fruits, vegetables and whole grains, has been associated with higher sleep quality and an increased microbial diversity of the gut microbiome profile, seen by increased growth of *Bifidobacteria, Bacteroides, Faecalibacterium prausnitzii* and SCFA-producing gut bacteria *Clostridium leptum* and *Eubactrium rectale*, whereas lower growth of *Firmicutes* and *Blautia* species.^205–207^ Individuals experiencing sleep deprivation have been shown to exhibit a distinct gut microbial profile compared to those with normal sleep patterns. Notably, there was an observed increased *Firmicutes:Bacteroides* ratio in those with sleep disturbance.^204^ Conversely, CKD is associated with sleep disorders like obstructive sleep apnea that can induce gut dysbiosis, as obstructive sleep apnea-related hypoxia alters gut wall permeability and promotes inflammation.^208^

The gut microbiome not only influences sleep patterns but also plays a role in mental and cognitive well-being. Studies have reported an association between mild cognitive impairment in older adults and altered gut microbiota, as evidenced by changes in microbial composition, with increase in *Bacteroides* and *Flavonifractor* and decrease in *Ruminococcus, Butricimonas* and *Oxalobacter*.^209,210^ Between 16–38% of CKD patients in stages 3 to 5 experience cognitive impairment, with renal function correlating with the development of cognitive impairment and dementia.^211,212^ The primary contributor to cerebral dysfunction in CKD appears to be small vessel cerebrovascular disease, as indicated by the Rotterdam study linking dementia to reduced cerebral blood flow and increased arterial stiffness associated with impaired executive functions.^213,214^ Cognitive impairment in CKD can also be influenced by dialysis modalities, particularly hemodialysis, which involves greater hemodynamic changes and may not effectively filter out all uremic toxins, potentially leading to persistent cognitive deficits.^215,216^ Gut microbiota variations have been observed in CKD patients undergoing hemodialysis and kidney transplantation.^217^ CKD can disrupt calcium homeostasis through concurrent hyperparathyroidism and vitamin D deficiency, potentially contributing to cognitive impairment. Hypercalcemia as a consequence can induce arterial calcification aligning with the vascular hypothesis.^218^ Distinct cortical neural synchronization in CKD dementia as opposed to Alzheimer’s one suggest unique cognitive domains affected by CKD.^219^

## Dietary impact on health and environment

Throughout life, spanning infancy, adolescence and adulthood, the gut microbiome undergoes dynamic changes influenced by various factors both from the environment and the host. These factors include maternal health, age, delivery method, feeding practices, immune status, dietary habits, alcohol consumption, drug usage, psychological factors, ethnicity, geographical location and smoking habits.^220,221^ The impact of these factors may vary between males and females, contributing to sex-specific differences in gut microbiota composition and therefore overall health outcome.

The strong association between diet, the gut microbiome, overall health and environmental burden implies that modifying our dietary choices can improve our well-being. Understanding these interactions can have implications for the development of personalized nutritional interventions in CKD patients and others. The interaction between the gut microbiome and disease can be linked to a cyclical relationship. Alterations in the gut microbiome such as those induced by sleep restriction, red meat or artificial sweeteners can potentially contribute to the development of certain pathologic conditions through increased concentration of uremic toxins like TMAO, IS, PCS and PAG in the circulation. Conversely, the presence of certain medical disorders like CKD may also lead to alterations in the composition of the gut microbiome. This bidirectional relationship between the gut microbiome and disease creates a feedback loop where changes in the microbiome can influence the uremic milieu and disease progression, while diseased states can in turn impact the composition of the gut microbiota. Understanding this interplay is indeed crucial for developing targeted interventions and therapeutic strategies aimed at modulating the gut microbiome to promote health and mitigate disease progression.

Today, 700 million of the planet’s people are malnourished, while 2 billion people are overweight.^222,223^ If we should feed the 10 billion people who will live on the planet in 2050, the need for food will increase by more than 50%. Since 2019, the number of people facing acute food insecurity has increased from 135 million to 345 million. Agriculture already uses 50% of the planet’s vegetated land areas and livestock farming accounts for two-thirds of all agricultural land and half of all greenhouse gases in the atmosphere originate from food production. Food is the single strongest lever to optimize human health and environmental sustainability.^2,224^ A radical restructuring of the food system is required for healthy people to live on a healthy planet. Recent research and the Eat Lancet commission show that the food that we eat today is neither good for our health or the environment.^2,225^ It is evident that the unhealthiest foods often have the highest environmental impact and dietary transitions toward greater consumption of healthier foods improve environmental sustainability.^226^ If we could increase our intake of plant-based diets, grains and fermented nutrients and limit the intake of red meat and likely artificial sweeteners, this could not only reduce the risk of burden of lifestyle diseases but also reduce greenhouse gas emission and increase the resistance against environmental threats, such as air and water pollution.^33,227^

In summary, the future challenges are multidimensional including environmental aspects, personal choices as well as advancements in health care providing aspects. Future studies should pay more comprehensive attention to how alterations in the microbiome can influence future health and if and how existing and future strategies related to dietary aspects could be successfully implemented to daily routines to ameliorate the burden of chronic diseases with the potential for novel treatment strategies.

## Data Availability

No data is needed.^23–25^
